# Combined exposure to night work and noise in relation to hyperglycemia among long-term night workers: a nationwide population-based prospective cohort study

**DOI:** 10.5271/sjweh.4215

**Published:** 2025-05-01

**Authors:** Po-Ching Chu, Chen-Hsien Lee, Yu-Fang Lee, Joyce Lin, Jui Wang, Jing-Shiang Hwang

**Affiliations:** 1 Department of Environmental and Occupational Medicine, National Taiwan University College of Medicine, Taipei, Taiwan.; 2 Department of Environmental and Occupational Medicine, National Taiwan University Hospital, Taipei, Taiwan.; 3 Institute of Labor, Occupational Safety and Health, Ministry of Labor, New Taipei City, Taiwan.; 4 School of Occupational Therapy, National Taiwan University College of Medicine, Taipei, Taiwan.; 5 Department of Statistics, University of Washington, Seattle, WA, USA.; 6 Institute of Epidemiology and Preventive Medicine, National Taiwan University, Taipei, Taiwan.; 7 Health Data Research Center, National Taiwan University, Taipei, Taiwan.; 8 Institute of Statistical Science, Academia Sinica, Taipei, Taiwan.

**Keywords:** interaction effect, night work

## Abstract

**Objectives:**

This study investigated the association between combined exposures and hyperglycemia incidence, as well as the dose–response relationship between the duration of night work and hyperglycemia among long-term night workers.

**Methods:**

In this prospective cohort study, 12 716 night workers from the nationwide population were recruited. Hyperglycemia incidence was based on the one-year change in fasting blood glucose levels. Occupational noise exposure was defined as exposure to 8-hour time-weighted average sound levels of ≥85 decibels. Personal factors, including body mass index, and work-related factors, like monthly night work duration, were assessed. Multivariable logistic and linear regression models were used to explore the association.

**Results:**

In the multivariate logistic analyses, each additional day of night work was associated with an increased risk of hyperglycemia [adjusted odds ratio 1.05, 95% confidence interval (CI) 1.02–1.07]. In the normal fasting glucose group, each additional day of night work was associated with a linear increase of +0.07% (95% CI +0.03% – +0.12%) in the change in fasting glucose levels, and noise exposure was associated with a linear increase of +1.34% (95% CI +0.55% – +2.12%) increase in fasting glucose levels. Furthermore, the population exposed to noise and working ≥10 days of night work had a significantly higher increase of fasting glucose levels (β +5.71%, 95% CI +4.48% – +6.95%), with significant interaction effects (P for interaction <0.01).

**Conclusions:**

The possible dose–response relationship between duration of night work and changes in fasting glucose levels was found. The combined exposure to night work and noise posed a higher risk for hyperglycemia than exposure to night work alone.

According to the International Diabetes Federation, 537 million adults had diabetes mellitus (DM) in 2021, and the number is predicted to increase to 643 million by 2030 (1). Diabetes imposes a heavy burden for individuals and society, including healthcare costs and loss of productivity caused by disability (2). Compared to workers without diabetes, workers with diabetes have higher odds of permanent or temporary disability (3), leaving paid employment, and early retirement (3, 4). Hyperglycemia based on fasting blood glucose levels was used as a surrogate marker to predict the possible development of diabetes. Thus, preventing new diagnosis of hyperglycemia is crucial and cost-effective for decreasing the burden of diabetes.

Regarding the risk factors of diabetes, non-occupational risk factors, such as family history and obesity, are well known, and occupational risk factors, such as night work (5–8), psychological stress (8), and noise (9, 10), are also indicated for working population. A study showed that the prevalence of night work among the employed workforce in Japan was increasing (13.3% in 1997, 17.8% in 2002, 17.9% in 2007, and 21.8% in 2012) (11). Additionally, Pan et al (5) demonstrated a clear relationship between night shift work and risk of diabetes, with hazard ratios for women with 1–2, 3–9, 10–19, and ≥20 years of night shift work being 1.03, 1.06, 1.10, and 1.24 after adjustment, respectively. Gan et al (12) reported an adjusted odds ratio (OR) of 1.09 for the association between ever exposure to shift work and diabetes risk. Due to the rising percentage of night workers and the high risk of diabetes among this population, assessing the dose–response relationship between the duration of night work and fasting blood glucose levels, which is a surrogate marker to predict the possible development of diabetes, may be helpful for the prevention of work-related diabetes.

Evidence suggests a link between environmental noise exposure and the risk of diabetes (9, 13, 14). Shin et al (14) demonstrated that every 10-decibel increase in long-term exposure to road traffic noise was associated with an 8% increased risk of incident diabetes. Sakhvidi et al (13) observed a 6% increase in the risk of diabetes per 5-decibel increase in noise exposure. Although workers with noise exposure might have a higher level of noise and a longer duration of exposure than the general population exposed to environmental noise, few studies have investigated the association between occupational noise exposure and the risk of diabetes (15). It is unknown whether workers with occupational noise exposure have more adverse effects – such as hyperglycemia, pre-diabetes, or diabetes – than the general population. Though Dzhambov et al (15) found no significant association between occupational noise and diabetes, some limitations, such as noise exposure assessment by self-report, were mentioned. Further prospective studies delving into the association between occupational noise and diabetes or hyperglycemia are warranted.

Although current evidence suggests that night work has a higher risk of diabetes, there has been very little research on the dose–response relationship between the duration of night work and fasting blood glucose level or the combined exposures to night work and occupational noise. The time trend of increasing fasting blood glucose levels may be an early indicator for the detection of possible diabetes; thus, hyperglycemia incidence-based on the one-year change in fasting blood glucose levels was used as a surrogate to predict possible diabetes incidence. To bridge the knowledge gap and provide information for the prevention of work-related diabetes in the workplace, the aims of the present study were to investigate the association between combined exposures to night work and occupational noise and hyperglycemia incidence. The present study hypothesized that combined exposures to night work and occupational noise increase the risk of hyperglycemia.

## Methods

### Data

In 2019, the Occupational Safety and Health Administration, Ministry of Labor, Taiwan, established the database of a nationwide long-term night work cohort for research purposes. Before conducting this study, the Administration anonymized the date. Long-term night work was defined as a work schedule in which, over the past year, an individual’s working time included >3 hours between 22:00–06:00 hours the next day, and this situation had accumulated for >6 months in total.

### Study design

This is a prospective cohort study of long-term night workers from 2019 to 2020 based on data from the nationwide database. In this study, we investigated the risk factors for the new-onset of hyperglycemia among long-term night workers with or without occupational noise exposure.

### Study population

**Figure 1:**
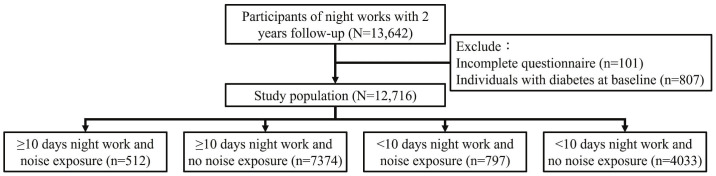
Flowchart of recruitment of the study population.

The first medical evaluation for workers with long-term night work was carried out in 2019, and the second medical evaluation was conducted in 2020. The mean follow-up duration was 348 days. The study population of the present study was all participants who fulfilled the definition of long-term night work and completed the two medical evaluations. A total of 13 642 participants were eligible. The exclusion criteria were participants with missing values for body mass index (BMI) and medical tests (N=101) at the first medical evaluation. The final sample included 12 716 participants after excluding those with hyperglycemia at baseline (N=807). The definition of hyperglycemia was fasting blood glucose ≥126 mg/dl or using antidiabetic drugs. The process of population selection is illustrated in figure 1.

### Outcome measures

In the present study, the incidence of hyperglycemia based on fasting blood glucose was used as surrogate to predict the possible incidence of diabetes. Based on the criteria for diabetes from the American Diabetes Association (16), the incidence of hyperglycemia was defined as fasting blood glucose ≥126 mg/dl or using antidiabetic drugs. Another outcome was the change in fasting blood glucose defined as the change from baseline (2019) to the following data (2020), divided by the baseline (2019) fasting blood glucose level. All participants were instructed to fast for ≥8 hours before collecting the morning blood samples and did not have the following conditions: critically ill conditions, obvious stress, injury, or trauma.

### Assessment of work-related risk factors

Participants who were exposed to occupational noise were defined as those who were exposed to an 8-hour time-weighted average sound levels of ≥85 decibels, as confirmed by environmental noise monitoring. Sound levels were measured using a precision sound level meter with A-frequency weighting and slow time weighting. The job tenure (in years) was assessed. Regarding the assessment of exposure to night work, the long-term night work characterized by a work schedule in which, over the past year, an individual’s working time included >3 hours between 22:00–06:00 hours the next day, and this situation had accumulated for >6 months in total was verified by human resources staff. Furthermore, the average monthly duration of night work (in days) was assessed through self-reporting. To evaluate the effect of combined exposure to night work and noise, the present study divided participants into four groups (i): participants with ≥10 days of night work in the past month and occupational noise exposure (N=512) (ii); participants with ≥10 days of night work and no occupational noise exposure (N=7374) (iii); participants with <10 days of night work and occupational noise exposure (N=797); and (iv) participants with <10 days of night work and no occupational noise exposure (N=4033).

### Assessment of associated variables

The following personal factors were assessed: age, sex, BMI, waist circumference, and medication history. The BMI was calculated as body weight (kg) divided by the square of height (m^2^). Medical tests were assessed, including systolic and diastolic blood pressure and blood tests. Hypertension was defined as systolic blood pressure ≥130 mmHg, diastolic blood pressure ≥80 mmHg, or receiving treatment for hypertension. The blood tests included fasting glucose, total cholesterol, triglycerides, low-density lipoprotein cholesterol (LDL-C), and high-density lipoprotein cholesterol (HDL-C). Dyslipidemia was defined as total cholesterol ≥200 mg/dL, triglycerides ≥150 mg/dL, LDL-C ≥130 mg/dL, HDL-C <40 mg/dL, or using lipid-lowering medication prescriptions (17, 18).

### Statistical analysis

We examined the baseline characteristics (ie, age, sex, BMI, blood pressure, total cholesterol, triglyceride, LDL-C and HDL-C, hypertension, dyslipidemia, job tenure, and average monthly duration of night work) among the four groups classified by the duration of night work and noise exposure. The descriptive results of continuous variables are expressed as the mean and standard deviation (SD), and the categorical variables are presented as numbers and percentages. The continuous and categorical data were compared between the four groups. An ANOVA test was used for continuous data, and Chi-squared test was used for categorical data. Logistic regression was used to identify factors associated with hyperglycemia incidence. We defined the dependent variable as equal to one when there was no hyperglycemia at baseline and hyperglycemia at followed-up, and zero otherwise. The independent variables considered in the analysis included night work, noise exposure, and four groups based on characteristics of night work and noise exposure. The univariable logistic regression model was first implemented for each independent variable separately. Multivariable logistic regression analysis was further performed to adjust for age, sex, BMI, hypertension, dyslipidemia, and baseline fasting glucose. To evaluate whether the effects of night work and noise exposure differed among participants with different baseline fasting glucose levels, we conducted stratified analyses. Participants were categorized into two groups: those with normal fasting glucose (<100 mg/dL) and those with pre-diabetes, defined as fasting glucose levels of 100–125 mg/dL (16). Furthermore, a linear regression model was applied to estimate the effect of night work and noise exposure on the changes in fasting blood glucose. A P-value of <0.05 was considered to indicate a statistically significant difference. All analyses were performed using SAS software version 9.4 (SAS Institute, Cary, NC, USA).

## Results

### Participant characteristics

The basic characteristics of the study population at the first medical evaluation are presented in table 1. The present study recruited 12 716 participants. The sex and age between the four groups were significantly different (P<0.01). In the group of ≥10 days of night work and no noise exposure, the majority were females (59.9%), while in other groups, the majority were males (P<0.01). The average age in the group with ≥10 days of night work was younger than that of the group with <10 days of night work (P<0.01). In particular, the group with ≥10 days of night work and noise exposure was the youngest (mean age of 34.8 years). The mean job tenure for participants with ≥10 days of night work was significantly shorter at <7 years compared to those with <10 days of night work, who had a mean job tenure >15 years (P<0.01). Comparisons of BMI, waist circumference, blood pressure, lipid profiles, hypertension, dyslipidemia, and fasting glucose levels among the four groups showed significant differences (P<0.01). The average monthly duration of night work differed significantly among the four groups (P<0.01). The mean duration was 16.9 days for the group with ≥10 days of night work and 7.8 days for the group with <10 days of night work.

**Table 1:** Descriptive statistics of the study population at the first medical evaluation, stratified by the duration of night work and noise exposure. [BMI=body mass index; SD=standard deviation]

		Total population (N=12 716)			Duration of monthly night work											P-value
					≥10 days						<10 days					
					Noise exposure (N=512)			No noise exposure (N=7374)			Noise exposure (N=797)			No noise exposure (N=4033)		
		Mean (SD)	N (%)		Mean (SD)	N (%)		Mean (SD)	N (%)		Mean (SD)	N (%)		Mean (SD)	N (%)	
Age		38.22 (10.69)			34.82 (7.72)			35.03 (8.86)			42.94 (10.71)			43.54 (11.57)		<0.01
Gender																<0.01
	Women		5296 (41.65)			41 (8.01)			4417 (59.9)			76 (9.54)			762 (18.89)	
	Men		7420 (58.35)			471 (91.99)			2957 (40.1)			721 (90.46)			3271 (81.11)	
BMI		25.09 (4.42)			25.06 (4.09)			24.85 (4.72)			25.07 (3.91)			25.53 (3.92)		<0.01
Waist		81.66 (11.19)			83 (10.41)			80.2 (11.67)			83.05 (9.96)			83.87 (10.15)		<0.01
Job tenure, years		10.12 (10.2)			5.85 (6.06)			6.97 (6.93)			16.12 (11.96)			15.21 (12.42)		<0.01
Fasting glucose (mg/dL)		89.4 (10.6)			89.93 (11.07)			87.85 (10.38)			91.23 (10.26)			91.82 (10.48)		<0.01
Blood pressure (mmHg)																
	Systolic	124.25 (15.64)			126.06 (14.56)			121.64 (15.08)			127.62 (16.2)			128.13 (15.73)		<0.01
	Diastolic	77.16 (11.93)			79.33 (10.38)			75.43 (11.14)			77.44 (12.81)			79.99 (12.72)		<0.01
Hypertension																<0.01
	Without	6496 (51.09)			230 (44.92)			4293 (58.22)			377 (47.3)			1596 (39.57)		
	With	6220 (48.91)			282 (55.08)			3081 (41.78)			420 (52.7)			2437 (60.43)		
Total cholesterol (mg/dL)		121.45 (102.52)			144.88 (101.3)			113.44 (99.15)			131.85 (123.28)			131.05 (102.8)		<0.01
Triglyceride (mg/dL)		192.35 (34.55)			188.73 (34.88)			188.98 (33.91)			196.11 (36.2)			198.24 (34.46)		<0.01
Low-density lipoprotein (mg/dL)		115.66 (31.76)			118.58 (32.54)			113.3 (31.69)			119.42 (32.6)			118.85 (31.27)		<0.01
High-density lipoprotein (mg/dL)		55.02 (13.85)			51.84 (12.34)			56.64 (14.29)			53.59 (13.16)			52.74 (12.89)		<0.01
Dyslipidemia																<0.01
	Without		5882 (46.26)			203 (39.65)			3793 (51.44)			311 (39.02)			1575 (39.05)	
	With		6834 (53.74)			309 (60.35)			3581 (48.56)			486 (60.98)			2458 (60.95)	
Noise exposure																<0.01
	Without		11 407 (89.71)			0 (0)			7374 (100)			0 (0)			4033 (100)	
	With		1309 (10.29)			512 (100)			0 (0)			797 (100)			0 (0)	
Average monthly duration of night work (days)		13.43 (5.5)				15.68 (5.06)			16.98 (4.01)			7.6 (0.59)			7.79 (0.72)	<0.01

### Univariable and multivariable analyses

During the one-year follow-up, 276 participants developed hyperglycemia. The univariable and multivariable analyses of the associations between the risk factors and hyperglycemia are presented in table 2. Although noise exposure demonstrated a significant risk in the univariable analysis [OR 1.54, 95% confidence interval (CI) 1.10–2.15], no significant relationship between noise exposure and hyperglycemia was observed after adjusting for confounding factors of age, sex, BMI, hypertension, dyslipidemia, and baseline fasting glucose (OR_adj_ 1.29, 95% CI 0.90–1.83). After adjusting for confounding factors, ≥10 days of night work emerged as a significant risk factor for hyperglycemia (OR_adj_ 1.84, 95% CI 1.38–2.44). Multivariable regression analysis also demonstrated that each additional day of night work significantly increased the risk of hyperglycemia (OR_adj_ 1.05, 95% CI 1.02–1.07, P<0.001). Furthermore, compared with participants who had <10 days of night work and no noise exposure, those with (i) ≥10 days of night work and no noise exposure, and (ii) ≥10 days of night work and noise exposure showed a significantly higher risk for hyperglycemia (OR_adj_ 1.82, 95% CI 1.33–2.47; OR_adj_ 2.78, 95% CI 1.64–4.71, respectively). Although the combined exposure to noise and ≥10 days of night work showed a greater effect than the sum of the two exposures, the interaction did not reach statistical significance (P for interaction=0.570).

**Table 2:** Risk factors associated with the incidence of hyperglycemia after one year follow-up, unadjusted and adjusted analyses. [OR=odds ratio; CI=confidence interval.]

		Incident hyperglycemia		Population		Incidence rate		Crude				Adjusted ^a^		
		N		N		%		OR	95% CI	P-value		OR	95% CI	P-value
Noise exposure														
	Without	235		11 407		2.06		1 (ref)				1 (ref)		
	With	41		1309		3.13		1.54	1.10–2.15	0.0123		1.29	0.90–1.83	0.1659
Duration of monthly night work														
	<10 days	110		4830		2.28		1 (ref)				1 (ref)		
	≥10 days	166		7886		2.10		0.92	0.72–1.18	0.5173		1.84	1.38–2.44	<0.0001
	Increasing 1 days							0.99	0.97–1.01	0.2094		1.05	1.02–1.07	0.0002
Combination of noise exposure and duration of monthly night work (≥10-day night work, noise exposure) ^b^														
	(No, No)	90		4033		2.23		1 (ref)				1 (ref)		
	(No, Yes)	20		797		2.51		1.13	0.69–1.84	0.6311		1.25	0.75–2.07	0.3927
	(Yes, No)	145		7374		1.97		0.88	0.67–1.15	0.3407		1.82	1.33–2.47	0.0001
	(Yes, Yes)	21		512		4.10		1.87	1.16–3.04	0.011		2.78	1.64–4.71	0.0001

^a^ Adjusted for age, sex, body mass index, hypertension, dyslipidemia, and baseline fasting glucose. ^b^ P for interaction=0.065. ^b^ P for interaction: crude=0.065; adjusted=0.570.

### Stratified analyses by fasting blood glucose

The results of the stratified analyses for participants with normal fasting glucose and those with pre-diabetes are presented in table 3. In the group of normal fasting glucose, participants with ≥10 days of night work had a 2.76-fold increased risk of incident hyperglycemia compared to those with <10 days night work (OR_adj_ 2.76, 95% CI 1.79–4.27). Similar to the total population, the combined exposure to noise and ≥10 days of night work resulted in a stronger effect than the individual exposures alone. However, this interaction did not reach statistical significance (P for interaction=0.447). In contrast, no statistically significant relationship was observed between noise exposure or the longest duration of night work and the incidence of hyperglycemia in the pre-diabetes group. Consequently, no interaction effect of combined exposure to noise and ≥10 days of night work on hyperglycemia was observed (P for interaction=0.865).

**Table 3:** Risk factors associated with the incidence of hyperglycemia after one year follow-up, unadjusted and adjusted analyses, stratified by fasting blood glucose. [OR=odds ratio; CI=confidence interval.]

			Crude				Adjusted ^a^		
			OR	95% CI	P-value		OR	95% CI	P-value
Normal fasting glucose (<100 mg/dL) (N=10 703)									
	Noise exposure								
		Without	1 (ref)				1 (ref)		
		With	1.54	0.94–2.52	0.0848		1.23	0.75–2.04	0.4142
	Duration of monthly night work								
		<10 days	1 (ref)				1 (ref)		
		≥10 days	1.59	1.07–2.37	0.0219		2.76	1.79–4.27	<0.0001
		Increasing 1 days	1.01	0.98–1.04	0.4882		1.05	1.02–1.09	0.0025
	Combination of noise exposure and duration of monthly night work (≧10-day night work, noise exposure) ^b^								
		(No, No)	1 (ref)				1 (ref)		
		(No, Yes)	1.06	0.44–2.57	0.9014		1.06	0.43–2.57	0.9049
		(Yes, No)	1.48	0.96–2.29	0.0786		2.61	1.62–4.20	<0.0001
		(Yes, Yes)	3.56	1.82–6.95	0.0002		4.19	2.10–8.36	<0.0001
Pre-diabetes (between 100 mg/dL and 125 mg/dL) (N=2013)									
	Noise exposure								
		Without	1 (ref)				1 (ref)		
		With	1.51	0.94–2.44	0.0889		1.24	0.73–2.08	0.4284
	Duration of monthly night work								
		<10 days	1 (ref)				1 (ref)		
		≥10 days	0.96	0.69–1.35	0.8280		1.24	0.82–1.87	0.2995
		Increasing 1 days	1.01	0.98–1.04	0.6080		1.04	1.00–1.08	0.0566
	Combination of noise exposure and duration of monthly night work (≧10-day night work, noise exposure) ^c^								
		(No, No)	1 (ref)				1 (ref)		
		(No, Yes)	1.50	0.82–2.76	0.1925		1.32	0.69–2.53	0.4106
		(Yes, No)	0.99	0.69–1.42	0.9381		1.28	0.83–1.98	0.2686
		(Yes, Yes)	1.51	0.69–3.27	0.3007		1.53	0.63–3.73	0.3452

^a^ Adjusted for age, sex, body mass index, hypertension, dyslipidemia, and baseline fasting glucose. ^b^ P for interaction: crude=0.132; adjusted=0.447. ^c^ P for interaction: crude=0.971; adjusted=0.865.

### Changes in fasting glucose and combined exposure in the normal group

**Figure 2:**
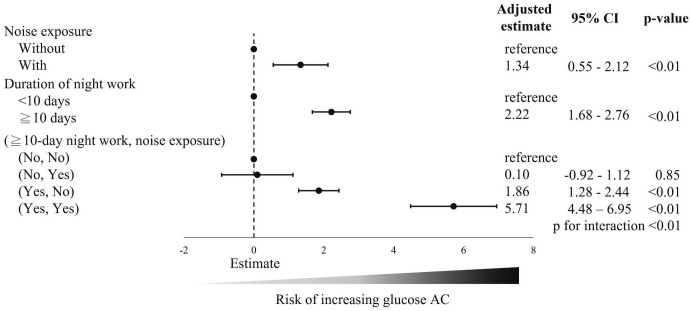
Risk factors associated with changes in fasting blood glucose after adjusted analysis in the normal fasting glucose group. a The change in fasting blood glucose defined as the change from baseline (first years) to the following data (second year) divided by the baseline (first years) fasting blood glucose level.

Figure 2 illustrates the risk factors associated with the change in fasting blood glucose after adjusted analysis in the normal fasting glucose group. The noise exposure group was associated with an increase of +1.34% (95% CI +0.55– +2.12%) in fasting glucose levels compared to the no noise exposure group. The group with ≥10 days of night work also exhibited a higher increase of fasting glucose levels compared to the group with <10 days of night work (β +2.22%, 95% CI +1.68– +2.76%). When analyzing the combined effects of noise exposure and night work, we found that compared to the reference group (no noise exposure and <10 days of night work), noise exposure with <10 days of night work did not lead to a increase in fasting glucose levels (β +0.10%, 95% CI -0.92– +1.12%). However, working ≥10 days of night shifts without noise exposure was associated with a significant increase in fasting glucose levels (β +1.86%, 95% CI +1.28– +2.44%). Notably, individuals exposed to both noise and ≥10 days of night work exhibited an even greater increase in fasting glucose levels (β +5.71%, 95% CI +4.48– +6.95%) compared to the reference group, with significant interaction effects (P for interaction=<0.01). This finding suggests that the combined exposure to noise and night work has an additive effect, resulting in a markedly higher increase in fasting glucose levels than either exposure alone.

In the normal fasting glucose group, the dose–response relationship between duration of night work and percentage changes in fasting glucose levels was found (supplementary material www.sjweh.fi/article/4215, figure S1). Specifically, each additional day of night work was associated with a higher increase of +0.13% (95% CI +0.08– +0.18%) in fasting glucose levels in the univariable analysis and a higher increase +0.07% (95% CI +0.03– +0.12%) in the multivariable analysis.

## Discussion

The present study indicated that after adjusting for confounding factors, night work for ≥10 days, compared with <10 days, emerged as a risk factor for hyperglycemia (OR_adj_ 1.84). Each additional day of night work was associated with an increased risk of hyperglycemia (OR_adj_ 1.05). Furthermore, in the normal fasting glucose group, each additional day of night work was associated with a 0.07% increase in percentage changes in fasting glucose levels. These results may support the possibility of a dose–response relationship between the duration of night work and fasting glucose levels. There have been several studies that have investigated the relationship between night work and diabetes; plenty of them have demonstrated that night work is a risk factor for diabetes (6, 19). A systematic review (6) summarized five epidemiological cohort studies that included large study populations. Most studies revealed that night workers have a higher risk of diabetes. Yohei Osaki et al (19) reported that shift work or night work had an obviously higher odds of diabetes after adjusted for confounders such as lifestyles, family history of diabetes, and BMI.

Regarding the possible mechanisms for the associations between night work and hyperglycemia, three potential explanations are as follows: (i) Compared to daytime workers, night workers are more likely to have altered eating behaviors, such as high-calorie diets (7), high-fat food diets (20), and nocturnal eating (21). Nocturnal eating may make digestion, absorption, and utilization of nutrients difficult (21). These eating behaviors may be risk factors for metabolic changes, which are risk factors for hyperglycemia (7). Therefore, altered eating behaviors may be potential mediators between night work and metabolic changes; (ii) In contrast to daytime workers, night workers tend to have lower levels of physical activity (22). Physical inactivity is a known risk factor for hyperglycemia (23). Therefore, night work may be associated with hyperglycemia through reduced physical activity; and (iii) night workers may be prone to sleep deprivation, which may alter cortisol levels (24) and melatonin profiles (25), thus leading to glucose intolerance and insulin resistance (7, 26).

The present study indicated that noise exposure group was associated with a 1.34% increase in fasting glucose levels compared to the no noise exposure group after adjusting for confounding factors in the normal fasting glucose group (β 1.34). Some research has revealed the relationship between environmental noise and an increased risk of diabetes (9, 14). Shin et al (14) showed that road traffic noise exposure was a risk factor for diabetes incidence. Additionally, Li et al (9) indicated an association between environmental noise exposure and metabolic syndrome, which generally has a high odds of developing into diabetes (27). Two other studies also indicated that an association between noise exposure and metabolic syndrome (28, 29). Interestingly, Li et al (9) found that occupational noise exposure posed a higher risk of metabolic syndrome compared to environmental noise.

The present study indicated that the population exposed to noise and working ≥10 days of night work had a significantly higher risk of increased fasting glucose levels compared to the reference group (no noise exposure and <10 days of night work) (β=5.71), with significant interaction effects (P for interaction <0.01). Tobías et al (30) explored the relationship between traffic noise (diurnal and night-time) and diabetes. Only night-time noise was significantly associated with the risk of mortality from diabetes. The study indicated a 9.4% increase in the risk of diabetes-related mortality with each 1 dB increase in night-time noise. Regarding the possible mechanism between combined exposure and diabetes, noise may excite the hypothalamic-pituitary-adrenal axis and the sympathetic-adrenal-medullary axis (9, 31, 32), inducing an increase in stress hormones such as cortisol (32). This, in turn, may promote the development of diabetes (9, 33). Vangelova found that workers on a counterclockwise rotating shift had high cortisol levels compared to those working permanent morning shifts (34). Zaman et al (35) discovered that high levels of noise, particularly at night, may cause endothelial dysfunction due to higher level of stress hormones, and Meigs et al (36) found that endothelial dysfunction predicts the development of diabetes. Furthermore, similar to the mechanism for association between night work and diabetes, noise exposure may also lead to physical inactivity (37) and change in eating behavior (13). Both physical inactivity and altered eating behaviors are known risk factors for diabetes (38, 39).

The present study indicated that in the group of workers with ≥10 days of night work per month and no noise exposure, the majority were females (59.90%). This finding is similar with a previous report, which found that 82.1% of current shift workers among electronics factories in South Korea were women (40). Variations in industry characteristics, tolerance to night work or noise exposure, and economic needs may affect the sex distribution of workers. Furthermore, sex differences regarding the relationship between night work and diabetes have rarely been studied (7, 41). Silva-Costa et al (7) indicated that night work affects men and women differently; increased obesity was obviously associated with night work in men, while increased fasting glycemia, glycated hemoglobin, and 2-hour plasma glucose were significantly associated with night work among women. Thus, further studies should explore the long-term health effects among specific populations, such as female workers with prolonged exposure to night work.

Several possible reasons may account for the age differences. Younger workers may have better health and thus be better able to tolerate exposure to night work or noise compared to older workers. Additionally, these high-risk jobs may have higher turnover rates. For example, younger individuals may have a better ability to adapt to circadian rhythm disruptions caused by night work, while older workers may be more likely to switch to jobs with better working conditions as they advance in their careers.

### Strengths and limitations

The present study had several strengths. First is the use of a nationwide database with a large sample size and a one-year follow-up design, which enhance the generalizability of the findings. Second, accurate fasting blood glucose levels were measured, and all data were obtained from participants who had fasted for ≥8 hours before collecting the morning blood samples and did not have the following conditions: critically ill conditions, obvious stress, injury, or trauma, ensuring the validity of the glucose measurements. Third, the definition of noise exposure was based on objective criteria, in which noise exposure was defined by an 8-hour time-weighted average sound level of ≥85 decibels, as confirmed by environmental noise monitoring, providing a reliable assessment of occupational noise exposure.

The present study had several limitations. First, as it was a one-year follow-up study, a longer follow-up period may be required to gain a more comprehensive understanding of the relationship between occupational noise and hyperglycemia in night workers. Second, due to the lack of information, the present study could not consider some confounding factors, such as family history of diabetes. Third, as is common in occupational epidemiology studies, it is necessary to be concerned about the healthy worker effect (15). Workers who had health problems may have left their jobs earlier. Fourth, due to unavailable detailed work schedule data, this study could not assess the intensity and characteristic of night shift work, including the number of consecutive night shifts, and the starting or ending times of the night shift.

### Concluding remarks

Through our study using the nationwide database, night work for ≥10 days, compared with <10 days, emerged as a risk factor for hyperglycemia, and a dose–response relationship between the duration of night work and fasting glucose levels was found. In the normal fasting glucose group, the population exposed to both noise and night work for ≥10 days had a higher risk of increased fasting glucose levels than those exposed to night work alone or noise exposure alone. Significant interaction effects support the possible combined effect of both exposures. This information may serve as a useful reference in the working environment to help design multifactorial intervention strategies aimed at reducing the risk of work-related hyperglycemia or diabetes. The development of a program for the early detection and prevention of work-related hyperglycemia or diabetes in this working environment is warranted. Future large-scale studies with longer longitudinal follow-up periods are needed to further elucidate the impacts of personal factors and work-related factors (eg, night work and noise exposure) on hyperglycemia or diabetes.

## Supplementary material

Supplementary material

## Data Availability

Data of this study are available from the Occupational Safety and Health Administration, Ministry of Labor, Taiwan for researchers who meet the criteria for access to confidential data, and the restrictions prohibit the authors from making the minimal data set publicly available.
