# Supplementary material for: Combined exposure to night work and noise in relation to hyperglycemia among long-term night workers: a nationwide population-based prospective cohort study
Source: Scand J Work Environ Health. 2025 Apr 27;51(3):237–46. doi: 10.5271/sjweh.4215 (PMC12072438; doi:10.5271/sjweh.4215)

Combined exposure to night work and noise in relation to hyperglycemia among long-term night workers: a nationwide population-based prospective cohort study[1]

by Po-Ching Chu, PhD, Chen-Hsien Lee, PhD, Yu-Fang Lee, BS, Joyce Lin, MS, Jui Wang, PhD,[2] ***Jing-Shiang Hwang, PhD***

1. Supplementary material
2. Correspondence to: Dr. Jui Wang, Institute of Epidemiology and Preventive Medicine, National Taiwan University, Taipei 100, Taiwan. [E-mail: juiwang92@ntu.edu.tw]

Supplementary figure.1 Relationship between monthly duration of night work and percentage changes in fasting glucose levels in the normal fasting glucose group

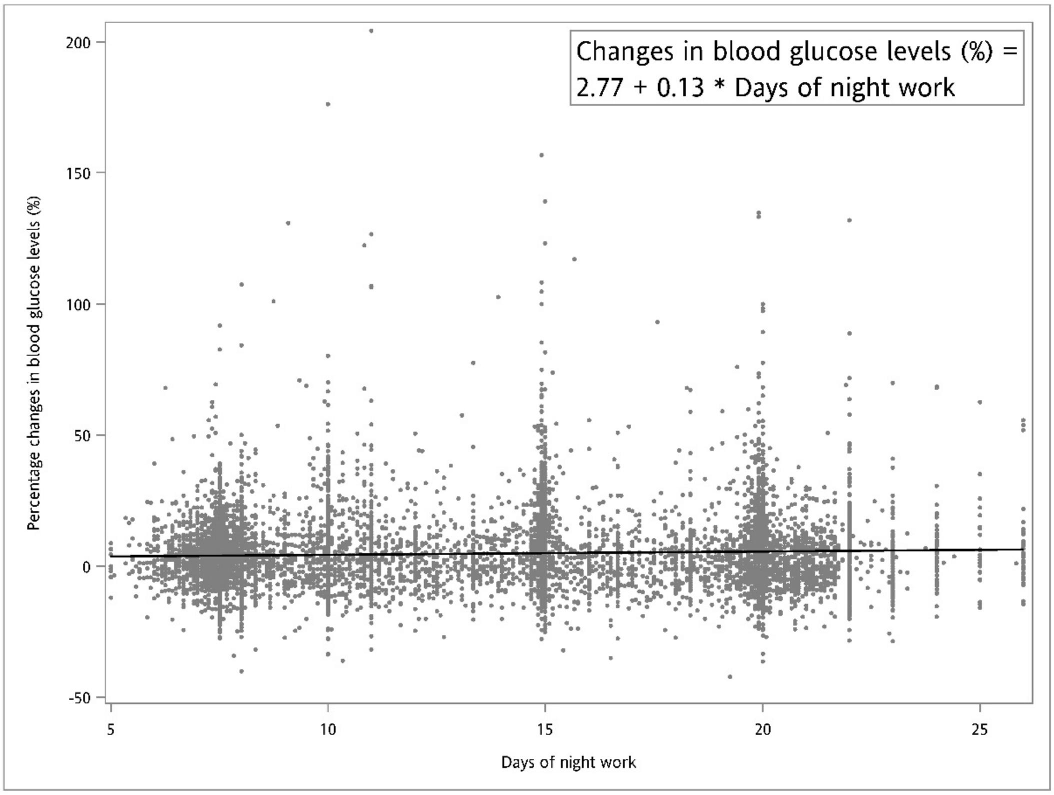

Supplement: Supplementary material [file SJWEH-51-237-S001.pdf]
